# Keas Perform Similarly to Chimpanzees and Elephants when Solving Collaborative Tasks

**DOI:** 10.1371/journal.pone.0169799

**Published:** 2017-02-15

**Authors:** Megan Heaney, Russell D. Gray, Alex H. Taylor

**Affiliations:** 1 School of Psychology, University of Auckland, Auckland, New Zealand; 2 Max Planck Institute for the Science of Human History, Jena, Germany; 3 Research School of the Social Sciences, Australian National University, Canberra, Australia; Utrecht University, NETHERLANDS

## Abstract

Cooperation between individuals is one of the defining features of our species. While other animals, such as chimpanzees, elephants, coral trout and rooks also exhibit cooperative behaviours, it is not clear if they think about cooperation in the same way as humans do. In this study we presented the kea, a parrot endemic to New Zealand, with a series of tasks designed to assess cooperative cognition. We found that keas were capable of working together, even when they had to wait for their partner for up to 65 seconds. The keas also waited for a partner only when a partner was actually needed to gain food. This is the first demonstration that any non-human animal can wait for over a minute for a cooperative partner, and the first conclusive evidence that any bird species can successful track when a cooperative partner is required, and when not. The keas did not attend to whether their partner could actually access the apparatus themselves, which may have been due to issues with task demands, but one kea did show a clear preference for working together with other individuals, rather than alone. This preference has been shown to be present in humans but absent in chimpanzees. Together these results provide the first evidence that a bird species can perform at a similar level to chimpanzees and elephants across a range of collaborative tasks. This raises the possibility that aspects of the cooperative cognition seen in the primate lineage have evolved convergently in birds.

## Introduction

Cooperation is one of the defining features of our species. Our ability to work together in groups played a key part in our evolutionary history and is crucial for the maintenance of our current societies [1–4]. The scope and complexity of cooperative behaviours exhibited by humans is thought to be due to a range of cognitive mechanisms, including those that allow us to understand cooperation [5] and motivate us to collaborate frequently [6].

Over the last two decades a large number of studies have searched for cooperative cognition in other species. The ‘loose string’ paradigm has emerged as the benchmark test for examining whether animals understand cooperation [7,8], and are motivated to collaborate [6,9]. In this test two animals can pull a platform baited with food rewards within reach if they simultaneously pull on both ends of a string; if only one end of the string is pulled the other end is pulled out of reach, making the baited platform inaccessible. A number of studies have demonstrated that animals can learn to pull the string simultaneously (e.g. [7,8,10–14]). Only a few of these species have been shown to pass delay tasks: dogs will wait 2 seconds for a partner, but not 15 seconds [14], while chimpanzees have been shown to wait 30 seconds [8] and elephants 45 seconds [10].

While these studies raise the possibility that these species have an understanding of collaboration, rather than simply using associative learning, only chimpanzees have been shown to flexibly change their behaviour towards the loose string task depending on whether collaboration is required or not [8], though coral trout have demonstrated similar flexibility in their natural hunting behaviours [15]. Interestingly, chimpanzees, despite appearing to understand collaboration, do not appear as motivated to cooperate as humans are. When given the choice between working with a conspecific and working on an asocial task, chimpanzees show no preference for either task [6], though if one extra banana is added to either option they will choose that task [9]. Children, in contrast, prefer to work together, even when no additional material reward can be gained from doing so [6], suggesting young humans have a unique motivation to collaborate with others.

Birds have been notably poor to date in their performances with the loose string paradigm, with both rooks and ravens failing to wait for a partner, or track whether collaboration is required or not [12,13]. African grey parrots have also struggled with these problems [11]. These results therefore suggest that there may be differences between bird and mammal species in their ability to understand cooperation. However, to date, only a small subset of bird species have been tested with problems requiring cooperation.

Here, we presented the kea (*Nestor notabilis*), a parrot species endemic to New Zealand, with the loose string task. This species lives in complex social groups and exhibits both extractive foraging behaviours and high levels of social play in the wild [16–18]. In captivity this species has been able to learn to use other individuals as a social tool and has performed well at tasks designed to test technical intelligence [19–22]. We presented keas with a series of five conditions, each designed to examine different aspects of their understanding of cooperation. Experiment 1 examined whether keas could coordinate their actions simultaneously on the loose string task. Experiment 2 tested whether keas could wait for their partner to be present in order to successfully complete the task, by delaying the arrival of the partnering kea in increasing increments. Experiment 3 tested whether subjects could flexibly alter their behaviour, depending on whether they needed to work with a partner or not. Experiment 4 tested if keas would attend to whether their partner had access to the string. In the final experiment we tested if keas had a prosocial bias for cooperation, in that they preferred to work with a partner to gain food, rather than work alone, when the reward for both tasks was the same.

## Materials and Methods

We tested four male kea, aged between one and three years. Neo and Zak formed the first pair and Bruce and Taz formed the second pair. Pairs were selected based on their preference for one of two apparatuses and whether they would tolerate the other keas inside the other side of the apparatus. Pairs remained fixed throughout testing. Keas were captive bred at Willowbank Wildlife Reserve in Christchurch with the exception of Bruce who was born in the wild and came to Willowbank as a fledgling. Subjects shared a large outdoor aviary with nine other keas and were free to come and go from the apparatus at any time. Food and water were available ad libitum within the aviary. This research was approved by The University of Auckland Animal Ethics Committee (approval AEC 001416).

### Apparatus and general setup

**Fig 1:**
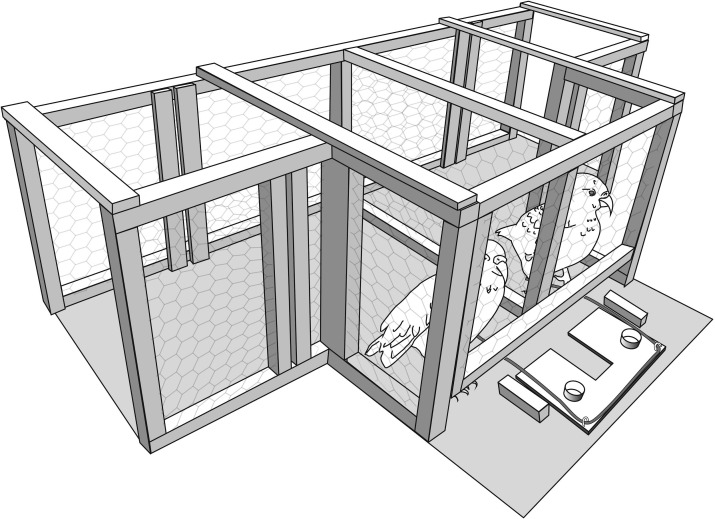
Drawing showing experimental setup for the simultaneous release experiment. Subject is presented with the duo platform which can only be pulled in with the help of a partner.

Subjects were trained and tested in a wooden apparatus (150 x 50 x 100 cm) (Fig 1). The apparatus had a wooden frame covered in chicken wire, which enabled subjects to see each other but not interfere with their partner's behaviour. The top of the apparatus was not covered so that subjects did not feel trapped or restricted and could exit the apparatus at any point. The apparatus could be entered at two separate entry points at opposite ends of the apparatus. Once inside neither kea could gain access to the other kea’s side as the apparatus was divided down the middle with a permanent wire partition.

A wooden platform (20 cm x 15 cm) with bottle tops glued to each side for rewards was placed just outside the wire partition in the middle of the apparatus. A piece of rope (45 cm) was threaded through two loopholes on the back left and right hand corner of the platforms. Pulling both ends of a piece of rope attached to the platform would cause it to slide through a small gap at the bottom of the apparatus and so allow retrieval of the rewards. To keep birds out of the apparatus when the experimenter was setting up or for experimental purposes, a wooden partition was placed just in front of each entry point of the apparatus. Each partition had large holes cut out of them so that the keas could still see inside the apparatus and view each other.

For experiments 1–3 only one side of the apparatus was used. The apparatus was enlarged and modified for Experiments 4–6 as they required subjects to choose between two platforms on either side of the apparatus. For this, the apparatus was simply doubled in size with the new side mirroring the existing side. Data was coded from a Panasonic 3MOs camera which was attached to a tripod overlooking the apparatuses beside the experimenter. Data was also manually recorded at the time of testing.

### Procedure

#### Familiarization and training

Keas were first habituated to entering the apparatus and receiving a reward from the experimenter under the small gap that the platform was placed behind. Each bird was then trained to pull both ends of the rope attached to the platform so that an individual bird could pull in the platform alone (solo apparatus). Around 5 cm of the rope was available to the kea for pulling each trial. Training to pull the platform in individually involved four stages. In the first stage both ends of rope overlapped, in the second stage they were placed 1 cm apart and in the third and fourth stages they were placed 3 cm and 6 cm apart respectively, as in past studies [23]. Once subjects achieved 3 successful trials in a row on stage 1 they moved on to the next stage. Three consecutive errors at any one stage meant that birds moved back to the previous stage. Training ended once each bird successfully completed stage four. The solo platform used for Bruce differed slightly. Bruce lost his upper mandible in an accident in the wild thus preventing him from being able to gather up two pieces of separated rope. Thus Bruce only received training with the two ends of the rope tied together. Keas received between half to one piece of Hills Science Diet (a reward not available to kea ad libitum) during each training and experimental trial.

#### Experiment 1: simultaneous release

The first experiment examined whether keas can work together by performing the same action simultaneously. The platform was placed out of reach of both subjects with one end of the rope available to each bird. The ends of the rope were 30cm apart and subjects had to wait to be released behind the wooden partitions. Partitions were removed and both birds were released into the apparatus simultaneously. Pairs received up to two sessions a day consisting of 20 trials in each session. Testing continued until each pair successfully pulled in the platform in 90% or more of trials in one session (18/20 trials).

#### Experiment 2: delayed partner release

**Fig 2:**
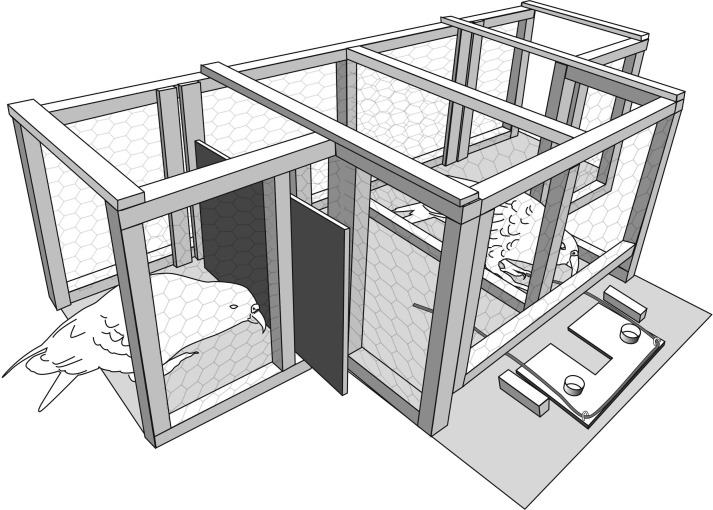
Drawing showing experimental setup for the delayed partner arrival experiment. Subject is presented with the duo platform which can only be pulled in when the partner arrives at the apparatus.

Keas first went through as shaping procedure, as used with elephants [10] and chimpanzees [8]. Here, partner arrival was initially delayed between one and two seconds (Fig 2). Once birds had individually succeeded in waiting for this delay period for three successive trials (as in [10]) the delay period was increased to 5 seconds. This increased by 5 second increments until each bird was successful at waiting for their partner for 25 seconds for three successive trials. Three successive failures at a given delay period meant that birds regressed back to the previous shorter delay period. Each bird was tested in sessions consisting of blocks of 10 trials as the focal bird for up to 5 sessions a day. Testing ended once a subject completed three successive trials at 25 seconds.

At test the keas were presented with 6 partner delay sessions consisting of 10 trials per session. Each block of ten randomized trials included three trials of partner delays ranging between 0–25 seconds, three trials consisting of previously unexperienced delays ranging between 26–45 seconds and 4 trials of unexperienced delays ranging between 46–65 seconds. This procedure was similar to the one used with elephants [10], except that we extended the maximum partner delay time by 20 seconds.

#### Experiment 3: solo and duo platforms with partner delays

Success at the simultaneous release task can be explained by individual action scaffolded by the structure of the task itself [12], while success at delay tasks can be explained by the learning of a combination of cues, such as seeing a partner while feeling tension on the rope [25]. To date, only one study has shown that an animal is able to flexibly alter its behaviour toward the loose string apparatus depending on whether collaboration is necessary or not. Melis et al. [8] showed chimpanzees would choose to allow a partner to enter a testing room more often when presented with an apparatus that required two individuals to solve it compared to when they could solve it by themselves. In contrast, studies with both rooks and parrots [11,12] have shown that birds typically fail to behave flexibly when presented with situations where cooperation either is, or is not, required. In Experiment 3 we created a similar situation to past studies: we presented either the duo or solo apparatus to subjects in a randomised order, and looked to see whether the keas would flexibly alter whether they waited for a partner to arrive before pulling the string, depending the apparatus type. Experiment 3 was comprised of an assessment phase and a test phase, as described below:

In order to run Experiment 3 we needed a delay period for the arrival of the partner that was sufficient for each bird to solve the solo condition, but not too long as to lead to the task becoming a test of inhibitory control, rather than cooperative cognition. We therefore set the delay period for each individual based on their performance at two assessment tasks. The first assessment task examined how long it took each bird to solve the solo condition. We gave birds 5 trials of the solo rope pulling condition, where the rope ends were placed 1cm apart, without a partner present in the apparatus, and recorded the average time taken to solve the task for each kea. The second assessment task examined whether each kea could inhibit pulling the string consistently when a partner was delayed by the time it took to solve the solo condition plus 3 additional seconds. Subjects therefore received 5 delay trials where they had to wait for their partner for the average time it took them to solve the solo condition and an additional three seconds. If subjects did not solve these two tasks in their first 10 trials (5 for solo, 5 for duo), testing continued until each kea successfully pulled the platform in for 5 consecutive trials of each condition within one session.

**Fig 3:**
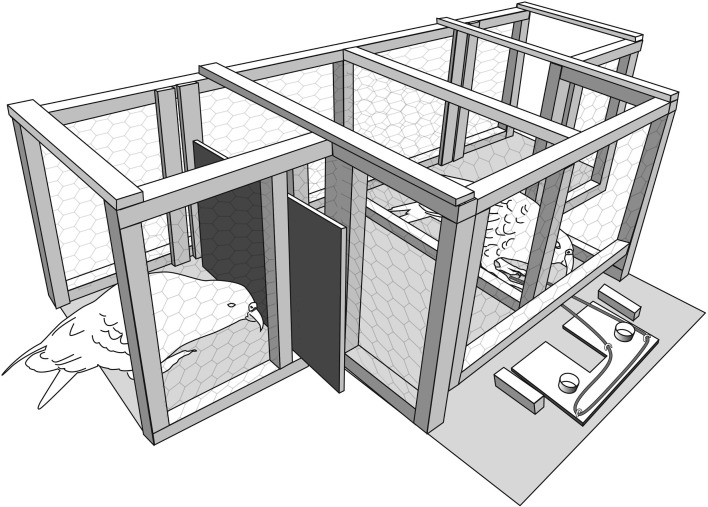
Example of the solo condition that was presented to subjects in Experiment 3. Subjects received an equal number of solo condition and delayed duo condition (Fig 2) trials in a pseudorandomized order.

At test Keas were presented with the same platform used in previous experiments. As in the assessment task, the platform presented was either the duo condition (one rope available to the focal kea and one to their partner) (Fig 1) or the solo condition (both ropes available to focal kea) (Fig 3)). The arrival of the partnering kea was delayed in every trial the average time it took each subject to successfully solve the solo condition with an added 3s. Thus keas had to choose between pulling the rope available immediately or waiting for their partner. Subjects received 10 trials a day for two days as the focal bird. The presentation order of the Solo and Duo platform conditions was pseudo-randomized across trials (no more than two of the same trials were presented in a row to avoid side biasing).

#### Experiment 4: no rope test

We presented the keas with the choice of opening a door to pull an apparatus where their partner had access to a rope on the other side of the loose string apparatus, or opening a door to an apparatus where the rope on the partner’s side was coiled and therefore out of reach, as in [10]. The rationale behind having the focal kea open a Perspex door was that it clearly indicated a choice by the kea and so could be used by the experimenter as a signal for when the partner should be let in.

A training stage was necessary before beginning Experiment 4 as it required subjects to learn to open a Perspex door. Stage 1 involved the keas individually entering the apparatus and retrieving a reward that was placed under the gap where the platform was usually accessed on either their left or their right hand side. The sides that the rewards were on were randomised. Keas moved to stage two once they had chosen the correct side for four trials in a row. Stage 2 was the same as stage one except that Perspex doors were placed on both sides of the apparatus. Keas had to choose the correct door to slide open and access the reward. Birds moved on to testing after completing four successful trials in a row.

Testing began with both keas waiting behind the wooden partitions. The focal kea was let in first. A platform with ropes available to both keas was placed on one side of the apparatus. An identical platform was placed on the other side of the apparatus except that while the focal keas rope was accessible the partnering keas rope was coiled up and out of reach. To access either of the platforms the focal kea had to open a Perspex door. There were no Perspex doors on the partnering keas side. Once the focal kea had made a choice by beginning to open a door, the partnering kea was let in. The partnering kea was let in after the subject to prevent the partnering kea from cueing the subject towards the platform with the partner’s rope available. The side that each platform was presented on was randomised and counterbalanced. Keas were not able to access the other platform once they had made their choice. Subjects received two sessions consisting of 10 trials each as the focal bird.

#### Experiment 5: solo/duo choice

Past work has shown that human children, but not chimpanzees are motivated to work together, rather than work alone for the same rewards [6]. We presented keas with a task that examined if they had a similar preference for working together.

Keas received a brief pre-training stage to preclude a possible side bias that may have developed for some subjects in Experiment 4. Two Perspex doors were set up on both the left and right hand side of the apparatus. Only one door had a reward behind it. Subjects were required to enter the apparatus and choose to slide a door on either their left or the right hand side. Training was done with a partner present. The side that the rewards were on were randomised and during half of the trials both the subject and partners’ rewards were on the same side and on the other half of trials rewards were on opposite side of their partner. This was done to rule out not only a bias to always choosing one side but also to rule out a bias towards going to the same or opposing side as their partner. Training stopped for each kea once they completed a minimum of 9/10 successful trials in a row.

At test we examined whether keas preferred to work alone (solo platform) or with a partner (duo platform) when rewards for both keas were the same on both platforms. Subjects had to choose between two platforms presented on opposite sides of the apparatus. One was a solo platform that could be pulled in individually on the focal kea’s side and the other was a duo platform that required the cooperation of a partner. The partnering kea was let into the apparatus first. To encourage the partnering kea to stand in front of the duo platform a wooden partition was placed over the partnering kea’s side that the solo platform was on so that the rewards on the solo platform were not visible to this kea. Thus the partner kea stood next to the duo apparatus. Both rewards were of course visible to the focal kea. While the solo platform and rewards were not visible to the partnering kea, if the focal kea chose to pull in the solo platform the partnering kea still received the reward through a gap between the bottom of the apparatus and the partition. When the partner was standing in front of the duo platform the focal kea was let in. Once the focal kea had chosen a platform and both keas had accessed the reward the partitions at the entry points of the apparatus were put in to prevent subjects from accessing the other platform. Each subject received one block of 10 trials for 2 days and they did not alternate roles until testing for the first kea was completed.

## Results

### Training to pull in the solo apparatus

It took Neo 80 trials (four sessions of 20 trials) to complete stage four of training. Taz reached criterion after 100 trials, Zak after 140 trials and Bruce after 180 trials.

### Experiment 1: simultaneous release

All kea reached criterion quickly. Neo and Zak scored 18/20 on their firstsession. Bruce and Taz scored 15/20 in session one, 17/20 in session two and then 20/20 in session three.

### Experiment 2: delayed partner arrival

During training All 4 keas learnt to wait for their partner in the delayed partner arrival condition (mean ± SD number of trials to reach criterion across all subjects = 97.25 trials ± SD 35.49, Table 1). Subjects made between 13 and 49 errors during this condition (mean ± SE across all subjects = 38 ± 16.79).

**Table 1:** Performance of the keas across Experiments 2–5. Trials (errors) show the total number of trials needed and errors made to reach criterion on the training stage of the delay experiment (Experiment 2). Randomized delay 0–65 secs and 26–65 secs show the number of successful trials keas completed at test in Experiment 2, both for all trials, and for trials longer than keas had previously experienced during training. Solo-Duo indicates the number of successful trials in Experiment 3, No Rope shows the same for Experiment 4, Prosocial Motivation shows the same for (Experiment 5 p < 0.05). Neo completed 40 trials of Experiment 5 because he was tested with two different kea.

Test	Taz	Neo	Zak	Bruce
Trials (errors) across Delay training	45 (13)	108 (44)	112 (46)	124 (49)
Randomised Delay 0–65 secs	55/60	49/60	53/60	45/60
Randomised Delay 26–65 secs	38/42	35/42	36/42	31/42
Solo/Duo	20/20	14/20	15/20	15/20
No Rope	14/20	9/20	9/20	10/20
Prosocial Motivation	11/20	32/40	7/20	11/20

At test, when presented with randomized partner delays of between 1–65 secs, subjects were successful between 75% and 92% of the time (mean ± SD across all subjects = 84 ± 7.41%, Table 1) (Fig 2). For the previously unexperienced delay periods ranging from between 26-65s all subjects were also successful at waiting for their partner’s arrival. This ranged from a success rate between 74% to 91% (mean ± SD across all subjects = 83 ± 7.10%).

### Experiment 3: solo/duo platform discrimination

In the assessment task Taz was successful in his first block of 10 assessment trials. Bruce took two blocks, Zak three blocks and Neo four blocks.

Across the two blocks of 10 test trials, three out of four subjects successfully demonstrated that they could discriminate between the solo and duo platforms at a significant level. That is, subjects chose to wait when presented with the duo platform, and immediately pull when presented with the solo. Taz scored 20/20 (Binomial choice, *p* = < 0.001), while Bruce and Zak both scored 15/20 (Binomial choice, *p* = 0.044), (Table 1). The only kea not to show a significant performance across both blocks, Neo, performed at chance levels in block 1 (5/10), and then significantly above chance in block 2 (Binomial choice,9/10 *p* = 0.027). All four subjects selected the correct course of action on the first trial of their first session.

### Experiment 4: no rope apparatus

No subjects were successful at discriminating between a duo platform with both ends of rope available to both keas and a duo platform with the partner’s rope coiled out of reach (See Table 1). Success at this task ranged between 45% and 70% (mean ± SD across all subjects = 53 ± 11.90%, Table 1).

### Experiment 5: solo duo platform choice test

Subjects selected the duo platform between 35% and 80% of trials (mean ± SD across all subjects = 56 ± 18.43%). Three of the keas did not show a preference for the duo apparatus (See Table 1). One subject, Neo had a significant preference for the duo platform (Binomial choice 16/20 trials *p* = 0.0139). We tested Neo again with a different partner to see if this preference was stable and he again showed a significant preference to work together for food, rather than alone (Binomial choice 16/20 trials *p* = 0.0139) (Table 1).

### Order effects

For all of the experiments, except Experiment 1 in which both keas served as subjects simultaneously, one kea in each dyad played the role of subject and one played the role of partner until testing was finished for that experiment. The roles were then reversed. However there is no evidence that presentation order affected our results. In Experiment 2, Neo served as subject first and reached criterion after 45 trials while his partner Zak, who went second, reached criterion after 108 trials. In the second dyad, Taz served as subject first and reached criterion in 112 trials while Bruce went second and reached criterion after 124 trials. For Experiment 3, Neo served as subject first and was successful in 70% of trials while Zak went second and was successful in 75% of trials. In the second dyad, Taz went first and was successful in 100% of trials while Bruce went second and scored 75%. No subject chose the correct platform in a significant amount of trials in Experiment 4 and in Experiment 5, Neo, who was the only subject who had a significant preference for collaboration, served as subject second in both dyads and so had previously only observed other kea choosing at chance between the duo and solo apparatuses.

## Discussion

Experiment 1 showed that, keas, like other birds and mammals [7,8,10–14] can coordinate their actions simultaneously with their partner to solve the loose string task. In the training stage of the delay experiment (Experiment 2) all subjects reached the 25s delayed partner arrival criterion, with one subject, Neo, making only 13 errors. For comparison, elephants made between 3–12 errors and chimpanzees made between 0–28 errors in a similar task [8,10]. At test, when presented with randomised delays, all subjects were able to wait for up to 65 seconds for their partner to arrive. This is longer than has previously been shown for any nonhuman species, though elephants and chimpanzees have been successful at waiting for 45 and 30 seconds respectively, and have not yet been tested on longer periods [8,10]. It should also be noted that the partner keas in the waiting task frequently shook the door in an effort to get to the string, as can be seen in our ESM, which possibly distracted the keas and so may have helped with waiting for such long periods.

In the solo/duo platform discrimination experiment (Experiment 3) all subjects were successful in differentiating between when they needed to wait for their partner to pull in the duo apparatus, and when they did not have to wait for their partner because they could pull in the solo platform by themselves. Only chimpanzees [8], and coral trout in natural foraging situations [15], have been shown to be able to discriminate between when they need a partner, and when they do not. In the no rope experiment (Experiment 4), no subject had a preference for the platform in which their partner’s rope was within reach, which is in contrast to the performance of elephants at this task [10]. Finally, in the solo/duo apparatus choice experiment (Experiment 5), three out of four keas did not have a preference for working with a partner. This is consistent with similar studies conducted with chimpanzees [6,9]. However, in a highly surprising result, one kea, Neo, did prefer to work with both a related and unrelated kea, rather than solve a task on his own, despite receiving no additional material benefit from collaborating. The same choices by children have been interpreted as evidence for humans’ having an intrinsic motivation to collaborate [6]. Neo’s behaviour therefore suggests at least one juvenile non-human animal also has this motivation to collaborate, though further controls still need to be run to rule out the possibility that Neo instead had a strong dislike for free-riding [6] or approached the duo apparatus due to stimulus or local enhancement [24]. It should be noted that this final possibility also applies to previous work on children [6].

As has been suggested in the past, simultaneously pulling the string, as in Experiment 1, may have occurred simply because subjects were released at the same time [12]. However, the small number of errors made by keas in the delay trials of Experiment 2, particularly by Neo who made only 13 errors, is comparable to the number of errors made by chimpanzees and elephants. The ability of keas to transfer to waiting for up to 65 seconds in the randomised delay trials impressive, given that this is 20 seconds longer than the longest delay period tested with elephants, and 35 seconds longer than that presented to chimpanzees. This performance suggests keas have excellent inhibitory control, despite their small absolute brain size [25–27]. In Experiment 2 keas had been rewarded for waiting until a partner had arrived before pulling the string. If they were using only associative learning to solve this task, they should have formed a rule such as “only pull when a partner is present”, or “pull when a partner is present and there is tension on the rope” (Seed & Jensen 2011). Experiment 3 directly tested this possibility by examining how keas reacted when they had to choose between waiting for a partner or immediately pulling the string, depending on the set-up of the apparatus. Keas clearly did not use the above associative rules: they pulled, rather than waited, when there was no partner, and no tension on the rope in the solo condition but did wait when there was no partner, and no tension on the rope, in the duo condition. The ability to flexibly alter cooperative behaviour depending on two factors, the presence of a partner and whether cooperation is required, is critical for effective cooperation. Only chimpanzees, and coral trout (in natural foraging situation) have been shown to flexibly alter their behaviours in this way [8,15]. Birds have typically failed at these tasks (e.g.[11,12]). Keas were given an initial assessment task in Experiment 3, which did give them additional experience of the solo and duo platforms. However, it is important to note that the amount of experience keas received during assessment was inversely related to their success at test. Taz, who passed the assessment task with no errors in the first 10 trials, went on to score 20/20 at test. Neo who received 40 trials of the assessment task, went on to score 5/10 in the first block of the test and 9/10 in the second. Taz flexibly altered his behaviour without making any errors across both the assessment and test tasks. This suggests that at least some keas had an understanding of how each apparatus worked and thus of when they did and did not need a partner, though it is clear that further work is needed to fully rule out the use of associative learning.

In the no rope experiment (Experiment 4) keas did not have a preference for the platform where their partner’s rope was within reach, rather than the platform where it was coiled up out-of-reach. One interpretation of this result, is that while the keas did have an understanding of when they needed a partner (as seen in Experiment 2), they did not have a clear idea of the role their partner played, in terms of interacting with the rope. This is supported by work suggesting that keas have some understanding of string connectivity or at least path continuity [21]. However, an alternate hypothesis is that the kea’s failure to differentiate between platforms in this condition may be due to other factors. In particular, understanding ‘connectivity’ seems to be highly difficult for birds, and is non-trivial for humans [28,29]. Due to this, the keas they may have understood that their partner’s role was to pull the string, but not been able to judge when the string was out-of-reach, due to their lack of a full causal understanding of how the string worked. That is, if an animal only understands that string allows another individual to take action at a distance, and so affect objects that are far away from it, such as the platform, a reasonable assumption is that this action at a distance can also take place on the string. Clearly future research is required to test between these possibilities. It is also important to note that while this result is in clear contrast to elephants, the only other species presented with the no rope experiment, it is not yet clear what elephants themselves understand of connectivity, as it is possible they used the combination of two associative cues (presence of partner and tension on the role) to solve this task [30].

Taken together, though our sample size was relatively low, our results do show that at least some keas can perform similarly to chimpanzees and elephants at a range of cooperative tasks. This suggests that aspects of the cooperative cognition seen in the mammalian lineage may have convergently evolved in at least one bird species. Given that keas engage in high levels of play and are both highly social and tolerant of conspecifics [16–18] it is possible that this type of group living may had led to the evolution this cognition. However, future research is clearly needed, both to show that the cognitive mechanisms used by the keas when solving the loose string task are the same as those seen in mammals, and to pinpoint the selective pressures that led to the evolution of this cognition.

## Supporting Information

S1 File Examples of kea performances across the experiment. (3GP)

## References

[1] Tomasello M, Melis AP, Tennie C, Wyman E, Herrmann E (2012) Two key steps in the evolution of human cooperation. Current Anthropology 53: 673–692.

[2] Henrich J, Henrich N (2007) Why humans cooperate: A cultural and evolutionary explanation: Oxford University Press.

[3] Nowak MA (2006) Five rules for the evolution of cooperation. science 314: 1560–1563. 10.1126/science.1133755 17158317 PMC3279745

[4] Boyd R, Richerson PJ (2009) Culture and the evolution of human cooperation. Philosophical Transactions of the Royal Society of London B: Biological Sciences 364: 3281–3288. 10.1098/rstb.2009.0134 19805434 PMC2781880

[5] Brownell CA, Ramani GB, Zerwas S (2006) Becoming a Social Partner With Peers: Cooperation and Social Understanding in One‐and Two‐Year‐Olds. Child development 77: 803–821. 10.1111/j.1467-8624.2006.00904.x 16942491 PMC3351034

[6] Rekers Y, Haun DB, Tomasello M (2011) Children, but not chimpanzees, prefer to collaborate. Current Biology 21: 1756–1758. 10.1016/j.cub.2011.08.066 22000101

[7] Hirata S, Fuwa K (2007) Chimpanzees (Pan troglodytes) learn to act with other individuals in a cooperative task. Primates 48: 13–21. 10.1007/s10329-006-0022-1 17103081

[8] Melis AP, Hare B, Tomasello M (2006) Chimpanzees recruit the best collaborators. Science 311: 1297–1300. 10.1126/science.1123007 16513985

[9] Bullinger AF, Melis AP, Tomasello M (2011) Chimpanzees, Pan troglodytes, prefer individual over collaborative strategies towards goals. Animal Behaviour 82: 1135–1141.

[10] Plotnik JM, Lair R, Suphachoksahakun W, De Waal F (2011) Elephants know when they need a helping trunk in a cooperative task. Proceedings of the National Academy of Sciences 108: 5116. 10.1073/pnas.1101765108 PMC3064331 21383191

[11] Péron F, Rat-Fischer L, Lalot M, Nagle L, Bovet D (2011) Cooperative problem solving in African grey parrots (Psittacus erithacus). Animal cognition 14: 545–553. 10.1007/s10071-011-0389-2 21384141

[12] Seed AM, Clayton NS, Emery NJ (2008) Cooperative problem solving in rooks (Corvus frugilegus). Proceedings of the Royal Society B: Biological Sciences 275: 1421–1429. 10.1098/rspb.2008.0111 18364318 PMC2602707

[13] Massen JJ, Ritter C, Bugnyar T (2015) Tolerance and reward equity predict cooperation in ravens (Corvus corax). Scientific reports 5. 10.1038/srep15021 PMC4595729 26442633

[14] Ostojić L, Clayton NS (2014) Behavioural coordination of dogs in a cooperative problem-solving task with a conspecific and a human partner. Animal cognition 17: 445–459. 10.1007/s10071-013-0676-1 23995845 PMC3920030

[15] Vail AL, Manica A, Bshary R (2014) Fish choose appropriately when and with whom to collaborate. Current Biology 24: R791–R793. 10.1016/j.cub.2014.07.033 25202866

[16] Diamond J, Bond AB (1991) Social behavior and the ontogeny of foraging in the kea (Nestor notabilis). Ethology 88: 128–144.

[17] Diamond J, Bond AB (1998) Kea, bird of paradox: the evolution and behavior of a New Zealand parrot: Univ of California Press.

[18] Diamond J, Bond AB (2004) Social play in kaka (Nestor meridionalis) with comparisons to kea (Nestor notabilis). Behaviour 141: 777–798.

[19] Tebbich S, Taborsky M, Winkler H (1996) Social manipulation causes cooperation in keas. Animal Behaviour 52: 1–10.

[20] Huber L, Gajdon GK (2006) Technical intelligence in animals: the kea model. Animal Cognition 9: 295–305. 10.1007/s10071-006-0033-8 16909237

[21] Werdenich D, Huber L (2006) A case of quick problem solving in birds: string pulling in keas, Nestor notabilis. Animal Behaviour 71: 855–863.

[22] Auersperg AM, Von Bayern AM, Gajdon GK, Huber L, Kacelnik A (2011) Flexibility in problem solving and tool use of kea and New Caledonian crows in a multi access box paradigm. PLoS One 6: e20231 10.1371/journal.pone.0020231 21687666 PMC3110758

[23] Seed AM, Tebbich S, Emery NJ, Clayton NS (2006) Investigating physical cognition in rooks, Corvus frugilegus. Current Biology 16: 697–701. 10.1016/j.cub.2006.02.066 16581516

[24] Gajdon GK, Fijn N, Huber L (2006) Limited spread of innovation in a wild parrot, the kea (Nestor notabilis). Animal cognition 9: 173–181. 10.1007/s10071-006-0018-7 16568276

[25] Jelbert S, Taylor A, Gray R (2016) Does absolute brain size really predict self-control? Hand-tracking training improves performance on the A-not-B task. Biology letters 12: 20150871 10.1098/rsbl.2015.0871 26843555 PMC4780546

[26] MacLean EL, Hare B, Nunn CL, Addessi E, Amici F, et al (2014) The evolution of self-control. Proceedings of the National Academy of Sciences 111: E2140–E2148. 10.1073/pnas.1323533111 PMC4034204 24753565

[27] Kabadayi C, Taylor LA, von Bayern AM, Osvath M (2016) Ravens, New Caledonian crows and jackdaws parallel great apes in motor self-regulation despite smaller brains. Open Science 3: 160104. 10.1098/rsos.160104 PMC4852647 27152224

[28] Taylor AH, Medina FS, Holzhaider JC, Hearne LJ, Hunt GR, et al (2010) An investigation into the cognition behind spontaneous string pulling in New Caledonian crows. PloS one 5: e9345 10.1371/journal.pone.0009345 20179759 PMC2825261

[29] Taylor AH, Knaebe B, Gray RD (2012) An end to insight? New Caledonian crows can spontaneously solve problems without planning their actions. Proceedings of the Royal Society B: Biological Sciences 279: 4977–4981. 10.1098/rspb.2012.1998 23097511 PMC3497243

[30] Seed AM, Jensen K (2011) Animal behaviour: Large-scale cooperation. Nature 472: 424–425. 10.1038/472424a 21525921

